# Lifetime prevalence, risk, and treatment of mood and anxiety disorders in Qatar's national mental health study

**DOI:** 10.1002/mpr.2011

**Published:** 2024-05-10

**Authors:** Salma Mawfek Khaled, Nour W. Z. Alhussaini, Majid Alabdulla, Nancy A. Sampson, Ronald C. Kessler, Peter W. Woodruff, Sheik Mohammed Al‐Thani

**Affiliations:** ^1^ Department of Population Medicine College of Medicine Qatar University Doha Qatar; ^2^ Department of Psychiatry Hamad Medical Corporation Doha Qatar; ^3^ College of Medicine Qatar University Doha Qatar Qatar; ^4^ Department of Health Care Policy Harvard Medical School Boston Massachusetts USA; ^5^ School of Medicine and Population Health University of Sheffield Sheffield UK; ^6^ Department of Public Health Ministry of Public Health Doha Qatar

**Keywords:** 5th edition (DSM‐5), diagnostic and statistical manual, lifetime prevalence, lifetime treatment, mental disorder, Qatar

## Abstract

**Objectives:**

To estimate lifetime prevalence, risk, and treatment for mental disorders and their correlates in Qatar's general population for the first time.

**Methods:**

We conducted a national phone survey of 5,195 Qatari and Arab residents in Qatar (2019–2022) using the Composite International Diagnostic Interview Version 3.3 and estimated lifetime mood and anxiety defined diagnoses. Survival‐based discrete time models, lifetime morbid risk, and treatment projections were estimated.

**Results:**

Lifetime prevalence of any disorder was 28.0% and was associated with younger cohorts, females, and migrants, but lower formal education. Treatment contact in the year of disorder onset were 13.5%. The median delay in receiving treatment was 5 years (IQR = 2–13). Lifetime treatment among those with a lifetime disorder were 59.9% for non‐healthcare and 63.5% for healthcare; it was 68.1% for any anxiety and 80.1% for any mood disorder after 50 years of onset. Younger cohorts and later age of onset were significantly predictors of treatment.

**Conclusions:**

Lifetime prevalence of mental disorders in Qatar is comparable to other countries. Treatment is significantly delayed and delivered largely in non‐healthcare sectors thus the need for increased literacy of mental illness to reduce stigma and improve earlier help‐seeking in healthcare settings.

## 1 INTRODUCTION

Mental disorders are recognized as a public health concern globally (Rehm & Shield, 2019; Vigo et al., 2016), affecting millions of individuals worldwide (World Health Organization, 2022). A meta‐analysis concluded that 30% of the general population suffer from a mental disorder at least once in their lifetime (Steel et al., 2014). In 2013, 5.4% of global Disability‐Adjusted Life Years and 17.4% of global years lived with disability were attributed to mental disorders (Vos et al., 2015). Notably, the peak age for incidence of mental disorder is around 15 years old (McGrath et al., 2023). Hence, the burden of these disorders across the lifetime is considerable (McGrath et al., 2023). Despite the significant burden and accompanying negative economic and psychosocial consequences of mental illness, treatment is often delayed or absent (Saxena et al., 2007; Wang et al., 2006).

In recognition of increasing interest in mental disorders worldwide, the World Health Organization (WHO) in collaboration with the World Mental Health (WMH) consortium conducted some of largest of epidemiological surveys of more than 150,000 participants across 29 countries. These studies use probability‐based sampling designs and uniform fielding procedures that allow comparison of estimates of prevalence and associations between participating countries (Kessler et al., 2018; ten Have, Tuithof, van Dorsselaer, Schouten, & de Graaf, 2023). The emerging data contribute significantly to our understanding of the global prevalence, burden, and treatment of mental disorders (Andrade et al., 2014; Evans‐Lacko et al., 2018; Harris et al., 2020; Kazdin et al., 2023; Kessler et al., 2018).

The primary disorders examined in the WMH surveys are major depressive disorders, bipolar disorders, dysthymic disorders, anxiety disorders, substance‐related disorders, and disruptive behavioral disorders (Kessler et al., 2018). These disorders are relatively prevalent in the participating countries with lifetime prevalence interquartile range (IQR) of 18.1%–36.1% (Kessler et al., 2009) and comprise 24.7% of the total population‐attributable risk proportion for disability (Alonso, 2012).

The WMH surveys do however highlight significant regional variations in the prevalence of mental disorders. Here, differences in prevalence of psychiatric disorders are associated with cultural factors as distinct from methodological differences (Alonso, 2012). To date, only three countries in the Middle East took part in the WMH survey initiative: Lebanon, Saudi Arabia, and Iraq. The lifetime prevalence of any mental disorder was highest in Saudi Arabia accounting for 34.2% followed by 25.8% in Lebanon, and 18.8% in Iraq (Alhasnawi et al., 2009; Altwaijri et al., 2020; Karam et al., 2008).

Mood and anxiety disorders were the most prevalent disorders in Lebanon (Karam et al., 2008) and Iraq (Alhasnawi et al., 2009), while anxiety and disruptive behavioral disorders were predominant in Saudi Arabia (Altwaijri et al., 2020). In contrast to Saudi Arabia, Lebanon and Iraq are countries that have experienced wars and political conflicts leading to declining economic status. The literature supports that populations exposed to political conflict had higher prevalence of certain mental disorders including MDD (Vos et al., 2015). According to a meta‐analysis, the Global Burden of Disease Study 2013, the age‐standardized pooled prevalence of MDD in conflict‐affected populations (7.6%) was two times higher than the global mean prevalence (3.5%) (Vos et al., 2015). Consistent with these findings is the reported observation of a higher lifetime prevalence of MDD in Lebanon (9.9%) and Iraq (7.2%) than in Saudi Arabia (6.0%).

Inadequate access to, and provision of, mental health services, adds the long‐term social and eventual financial burden associated with mental disorders across the world (Bristow et al., 2011; White, 2008). Prolonged periods without treatment or treatment delay, often years after the onset of illness, all contribute to reduced quality of life and poor clinical outcome (Reardon et al., 2017). Data from 15 developed countries that adopted the WMH surveys showed that respondents with any lifetime mental disorder who initiated immediate treatment contact in the year of onset ranged widely, for example, between 6.0% and 52.1% for mood disorders and 0.8% and 36.4% for anxiety disorders (Wang et al., 2007). The median delays among people with mood and anxiety disorders who sought treatment contact ranged from 1 to 14 years and 3 to 30 years, respectively. Those with mood disorders experienced shorter delays in treatment contact compared to those with anxiety disorders, possibly because they present with more severe symptoms that attract attention (Wang et al., 2007). Those with anxiety disorders experienced longer delays in receiving treatment in developing countries than in developed countries, possibly as these are considered “minor” and less in need of care than other health problems (Wang et al., 2007).

The extant literature on the treatment of mental disorders in various Arab countries points out that unfulfilled needs in psychiatric treatment is common and the majority of those who seek treatment for mental health problems receive treatment from the general medical sector rather than from the mental health specialty sector (Nasser & Salamoun, 2011). The lifetime prevalence of treatment in Saudi Arabia was 28.3% for those with any lifetime disorder: 33.0% for any anxiety and 35.4% for any mood disorders (Al‐Subaie et al., 2020). This was a noticeable low treatment prevalence rate when compared to other participating WMH countries (Al‐Subaie et al., 2020) underscoring the need to step up efforts to treat mental disorders in the Middle East.

The World Mental Health Qatar (WMHQ) is the first national population‐based epidemiological study of mental disorders in Qatar. In an earlier part of this issue, we explained the overall aim of the study along with providing an overview of Qatar's mental health service (Khaled, Al‐Abdulla, et al., 2024). The study was conducted through a collaboration between the Social and Economic Survey Research Institute (SESRI) at Qatar University, Hamad Medical Corporation (HMC), the Ministry of Public Health, the World Health Organization (WHO), Harvard Medical School University, and University of Michigan. Despite earlier studies carried out with Qatari nationals only in primary care setting using the WHO‐Composite International Diagnostic Interview Version 3.0 (Bener et al., 2015; Ghuloum et al., 2014), up to this point, there are no baseline population‐based lifetime prevalence and treatment estimates for common mental disorders in the country. Our study aimed to estimate the lifetime and treatment of mood and anxiety disorders and their correlates in the general population of Qatar. Hence, the findings from this study will contribute to our understanding of the epidemiology of mental illness in Qatar's nationals and Arab expatriates for the first time.

## 2 METHODS

### 2.1 Sample

Earlier in this issue, we described in details the sampling design and procedures used in the WMHQ survey (Khaled, Amro, Bader, et al., 2024). Using a national‐level cellular telephone frame prepared by SESRI with the help of the main telecommunications provider in the country, disproportionate stratified sampling was used to draw a representative sample of Arabic‐speaking adults 18 years or older who lived in Qatar during the survey reference period (January 2019 and January 2022). Respondents were contacted with a Short Message Service text prior to being surveyed using a remote Computer Assisted Telephone Interviewing (CATI) system. During 234 days of data collection, a total of 5195 interviews were completed in three waves (pilot, wave I, and wave II). All interviews were conducted in Arabic with the majority of interviews conducted with non‐Qataris Arabs (72.2%). The average length of the survey was 77 min (63.2–94.0). The average response rate was 19% and varied across survey waves from 17% to 26%.

### 2.2 Field procedures

Training, survey administration, and quality control procedures are described in detail in an earlier manuscript published in this issue (Khaled, Amro, Bader, et al., 2024).

#### 2.2.1 Ethics

The survey component of the WMHQ study was approved by Qatar University's ethics committee (QU‐IRB 1219‐EA/20). Survey procedures as well as study aims were explained verbally to the participants over the phone. Before beginning the telephone interview, verbal consent was obtained from each eligible subject who wished to participate in the study using a consent script that was included as part of the introduction section to the survey's interview instrument. All participants were aware of their right to stop or withdraw from the study at any point during the interview.

#### 2.2.2 Short‐ and long‐interview (part I and part II)

The survey was designed so that only those who met the minimum criteria of lifetime mental illness were required to complete the long‐form (part II) of the interview, while everyone else competed the short version (part I). This was achieved using an algorithm that divided all respondents into one of three groups at the end of the short interview for the WMHQ instrument: (1) those who we considered to have “threshold” disorders, (2) those who had “sub‐threshold disorders” and (3) all others. The algorithm selects 100% of the people in the first group, 50% of those in the second group, and 25% of the people in the third group into the long form. Weights were added to the final dataset to adjust for the under‐sampling of respondents in the second and third groups so that the weighted prevalence of disorders in the final sample had the same expected value as in the total original sample. These procedure are described in detail elsewhere in this issue (Khaled, Amro, Bader, et al., 2024).

### 2.3 Measures

#### 2.3.1 Demographic variables

Sociodemographic correlates included cohort (defined by age at interview in categories 18–29 years, 30–39 years, 40–49 years, and 50+ years), gender, and marital status (currently married, previously married, and never married). Highest education achieved by age at interview was defined as low (none, primary or preparatory school), low‐average (secondary school), high‐average (2‐year diploma), and high (bachelor's degree or more). Time varying migration status using year of migration (born in Qatar vs. age moved to Qatar) was used as a predictor in our lifetime models.

#### 2.3.2 Lifetime prevalence of mental disorders

The WMH‐Composite International Diagnostic Interview is a fully structured lay interview (Kessler et al., 2009; Kessler & Ustun, 2004) that generates diagnoses according to international diagnostic criteria for mental disorders. We used the latest version (3.3) of the Composite International Diagnostic Interview (CIDI) or CIDI‐5 which is based on the criteria in the Diagnostic and Statistical Manual of Mental Disorders, 5th Edition or DSM‐5 (American Psychiatric Association, 2013). Lifetime disorders considered here included anxiety disorders (panic disorder (PD), generalized anxiety disorder (GAD), post‐traumatic stress disorder (PTSD), and obsessive‐compulsive disorder (OCD)) and mood disorders (MDD, broad bipolar disorder I/II (BPD)). Post‐traumatic stress disorder and OCD were both assessed in part II of the survey and were included under the broadly defined anxiety disorder category in our analysis because they have traditionally been classified as anxiety disorders even though are now included in new separate categories in the DSM‐5.

As described in another manuscript published in this journal issue (Khaled, Amro, Abdelkader, et al., 2024), blind clinical re‐interviews using the Structured Clinical Interview for DSM‐5 (SCID) (First et al., 2015) found generally good concordance between WMH‐CIDI‐5 diagnoses and SCID diagnoses (AU‐ROC from 0.67 to 0.81). Comorbid disorders were defined as the number of disorders, exactly 1, exactly 2, and 3 or more. To assess the age of onset (AOO) for each disorder, respondents were asked to report the age when they first met criteria for the disorder and not the first symptom of the disorder.

#### 2.3.3 Lifetime treatment

All respondents who completed part II of the survey were asked about ever receiving treatments for their problems with emotions, nerves, or mental health. Treatment variables were created using questions detailing the services received from specific providers, which included Mental Health Hospitalization (hospitalized overnight for problems with emotions, nerves, or mental health), Mental Health Specialist (such as psychiatrist, psychologist, mental health counselor, social worker, marriage or family counselor, or psychiatric nurse), General Medical Professional (general medical doctor, nurse, or other general medical care provider), Spiritual Advisor (Imam, minister, priest, healer or other spiritual advisor), and Complementary and Alternative Medicine (CAM; self‐help or support group not lead by a mental health professional). Service providers were then collapsed into the following groups to capture specific treatment sectors: any healthcare services (Mental Health Hospitalization, Mental Health Specialist, or General Medical Professional), non‐healthcare services (Spiritual Advisor or CAM), and any treatment sector (healthcare and non‐healthcare). Respondents who reported ever seeking treatment from any of these providers were asked a follow‐up question about the age when they first sought treatment. Responses to this question were used to define the age of first treatment contact.

## 3 ANALYTICAL PROCEDURES

### 3.1 Lifetime prevalence analysis

Lifetime prevalence was estimated as the proportion of respondents who ever had a given disorder up to their age‐at‐interview. Projected lifetime morbid risk at age 65 was estimated using the Kaplan–Meier method implemented in SUDAAN 11.0.4 (Research Triangle Institute, 2020) whereas selected AOO percentiles were estimated using the life‐table method (also called the actuarial method) implemented in SAS 9.4 (SAS Institute, 2016). The actuarial method differs from the more familiar Kaplan–Meier method (Kaplan & Meier, 1958) in using a more accurate way of estimating the timing of onsets within a given year (Halli & Rao, 1992) but, like the Kaplan–Meier method, assumes constant conditional risk of onset at a given year of life across cohorts.

Predictors of lifetime prevalence were examined using discrete time survival analysis with a logistic link function and person‐year treated as the unit of analysis (Efron, 1988). As noted above in the description of sociodemographic correlates, all the variables other than gender were coded as time‐varying variables so as to estimate the associations of these variables with the subsequent first onset of each disorder. We applied this method separately for each of the disorders assessed in the WMHQ and we then pooled results across individual disorders to estimate three composite prediction equations for any anxiety, any mood, and any anxiety or mood disorders. The pooled models were based on combining the person‐year datasets created for each disorder separately for a given category into a single stacked dataset. This dataset included dummy predictor variables to distinguish the component datasets for the individual disorders. We then estimated pooled within‐disorder associations of the sociodemographic variables with the outcomes (i.e. first onset of a given disorder) across disorders based on the simplifying assumption that the odds‐ratios (ORs) were constant across disorders. Significance tests were carried out to evaluate the accuracy of that assumption so as to ascertain when to look at disorder‐specific ORs rather than summary ORs pooled across all disorders in the class.

Standard errors of prevalence estimates and logits were obtained using the Taylor series linearization method implemented in SAS 9.4 (SAS Institute, 2016). Standard errors of projected lifetime risk estimates were obtained using the Taylor series linearization method implemented in SUDAAN 11.0.4 (Research Triangle Institute, 2020). Logits and logits +/−2 standard errors were exponentiated to produce ORs and corresponding 95% confidence intervals (95% CIs). Multivariate significance tests of predictor sets were made with Wald *χ* ^2^ tests using Taylor series design‐based coefficient variance–covariance matrices. Statistical significance was evaluated consistently at the 0.05 level with two‐sided tests.

### 3.2 Lifetime treatment analysis

Projected cumulative lifetime probability of treatment over the 50 years post disorder onset was computed using the actuarial method for estimating survivor functions. The age of first treatment for any disorder defined the event, with censoring occurring at the age of recency of the disorder. After the survivor function was estimated, the cumulative probability of failure (CDF) was calculated from the survivor function. The CDF was then scaled to have a maximum value of 100, by dividing it by the cumulative probability of receiving treatment before the end of the 50‐year window. Predictors of lifetime treatment were examined using discrete time survival analysis with a logistic link function and person‐year unit of analysis (Efron, 1988). We did this separately for each of the assessed disorders. Then, as with prevalence, we estimated separate models for all respondents with any disorder in a given category beginning with AOO of the first disorder and defining initial treatment as the age when treatment first occurred for any disorder. ORs with corresponding 95% CIs and design‐adjusted Wald *χ* ^2^ tests of joint linear hypotheses were calculated. Statistical significance was evaluated consistently at the 0.05 level with two‐sided tests. All analyses were conducted using SAS version 9.4 (SAS Institute, 2016).

## 4 RESULTS

### 4.1 Sample description

The sample was recruited from a national telephone frame as described in an earlier paper published in this issue (Khaled, Amro, Bader, et al., 2024). Description of the sample characteristics is provided in Table 1. The mean age was 39 years (SE = 0.2). The sample included 39.2% females, 52.4% with high education (Bachelor's degree or higher), 21.9% were never married, and 35.9% never migrated (27.8% were Qataris and 8.1% were Arabs who were born and lived in Qatar all their life).

**TABLE 1:** Demographic variables in the World Mental Health Qatar (WMHQ) sample of people aged 18 years or older (*N* = 5195) in unweighted frequencies (*n*) and weighted percentages (%).

Demographics^a^	Total		
	*n*	%	SE
Gender			
Female	1980	39.2	0.7
Male	3215	60.8	0.7
Age at interview			
18–29	1085	22.3	0.7
30–39	1672	30.7	0.6
40–49	1310	24.3	0.6
50+	1128	22.7	0.7
Education^b^			
Student	17	0.4	0.1
Low	590	11.5	0.5
Low‐average	1310	26.1	0.5
High‐average	510	9.6	0.4
High	2768	52.4	0.7
Marital status			
Ever married	4092	78.1	0.6
Never married	1103	21.9	0.6
Migration status			
Migrated	3472	64.1	0.7
Never migrated	1723	35.9	0.7

Abbreviation: WMHQ, World Mental Health Qatar.

^a^ Variables defined at the age of interview.

^b^ Low = Preparatory School or less; Low‐average = Secondary School; High‐average = 2‐year diploma; High = Bachelor's degree or more.

### 4.2 Lifetime prevalence & sociodemographic correlates of mental disorders

The lifetime prevalence of any DSM‐5 mood or anxiety disorder in Qatar was 28.2% (Table 2). Many participants with any lifetime disorder also had multiple disorders. While 28% met the criteria for at least one disorder, slightly more than 11% met the criteria for at least two, 5% met the criteria for at least three and just over 1% met the criteria for four or more disorders. In fact, approximately 41% of all those with at least one disorder had two or more disorders (11.6%/28.2%), 41% of all those with at least two disorders had three or more disorders (4.7%/11.6%), and approximately 30% of those with at least three disorders had four or more disorders (1.4%/4.7%).

**TABLE 2:** Lifetime prevalence of DSM‐5/CIDI disorders in total sample and stratified by age and gender.

Specific disorder/disorder category	Qatar			Age categories								Test	Gender categories				Test
	Total			18–29		30–39		40–49		50+		Statistics	Female		Male		Statistics
	*n*	%	SE	%	SE	%	SE	%	SE	%	SE	$\chi_{3}^{2}$ (*p*)	%	SE	%	SE	$\chi_{1}^{2}$ (*p*)
Panic disorder	123	2.5	0.2	3.0	0.6	3.2	0.4	2.1	0.4	1.5	0.3	4.3 (0.009)*	3.6	0.4	1.8	0.3	15.1 (<0.001)*
Generalized anxiety disorder	287	5.7	0.3	7.0	0.8	6.6	0.6	4.9	0.7	3.8	0.6	4.6 (0.007)*	7.7	0.6	4.4	0.4	24.5 (<0.001)*
PCL‐SC PTSD^a^	731	18.2	0.9	22.7	1.6	20.6	1.5	16.9	1.6	11.4	1.5	8.2 (<0.001)*	23.9	1.4	14.5	0.9	39.8 (<0.001)*
Obsessive‐compulsive disorder^a^	134	2.9	0.3	3.3	0.7	4.1	0.6	1.8	0.5	2.0	0.6	4.3 (0.009)*	3.8	0.5	2.3	0.3	6.7 (0.012)*
Any anxiety disorder^a^	883	21.5	0.9	25.6	1.7	24.7	1.6	20.3	1.7	13.7	1.5	10.0 (<0.001)*	27.8	1.5	17.2	1.0	38.1 (<0.001)*
Major depressive disorder	614	12.0	0.5	17.3	1.2	13.4	1.0	11.0	1.1	6.0	0.7	27.2 (<0.001)*	17.3	0.9	8.6	0.6	70.7 (<0.001)*
Broad bipolar disorder	269	5.3	0.3	6.1	0.7	6.7	0.7	5.2	0.6	2.6	0.4	10.0 (<0.001)*	5.5	0.5	5.1	0.4	0.30 (0.611)
Any mood disorder	883	17.3	0.5	23.4	1.4	20.1	1.1	16.2	1.3	8.6	0.9	28.0 (<0.001)*	22.8	0.9	13.7	0.6	57.6 (<0.001)*
Any disorder^a^ (one or more)	1185	28.2	1.0	34.6	1.9	31.7	1.7	26.7	2.0	17.9	1.8	12.5 (<0.001)*	35.5	1.7	23.3	1.0	44.2 (<0.001)*
Two or more disorders^a^	557	11.6	0.5	16.2	1.3	13.7	0.9	9.5	1.0	6.1	0.8	15.4 (<0.001)*	16.0	0.9	8.7	0.6	52.2 (<0.001)*
Three or more disorders^a^	229	4.7	0.3	6.1	0.8	5.9	0.6	3.2	0.5	3.4	0.7	6.5 (<0.001)*	7.0	0.6	3.3	0.3	31.5 (<0.001)*
Four or more disorders^a^	71	1.4	0.2	1.7	0.4	1.7	0.4	1.0	0.3	1.3	0.4	1.4 (0.249)	2.1	0.4	1.0	0.2	7.6 (0.008)*
Sample size: Part 1	5195			1085		1672		1310		1128		‐‐‐	1980		3215		‐‐‐
Sample size: Part 2	2583			605		878		647		453		‐‐‐	1076		1507		‐‐‐

*Note*: Results reflect weighted data, with 50 strata and 100 clusters.

^a^ Disorder was only assessed in the Part 2 sample (*n* = 2583).

*Significant age difference at the 0.05 level.

Also shown in Table 2, the prevalence of any anxiety disorder (21.5%) was slightly higher than the prevalence of any mood disorder (17.3%). Among the individual disorders that were assessed, the most prevalent was PTSD at 18.2%, followed by MDD at 12.0%, GAD at 5.7%, BPD at 5.3%, OCD at 2.9%, and finally PD at 2.5% (Table 2).

Results from the stratified analysis of lifetime prevalence by age and gender are shown in Table 2. A significant trend of decreasing lifetime prevalence with increasing age at the time of interview was observed: the smallest decrease was for PD and OCD, while the largest decrease was for MDD and PTSD. Multimorbidity also significantly decreased with age at the time of interview except for those with four or more lifetime disorders (Table 2).

Gender differences across all disorders were statistically significant with higher lifetime prevalence among females than males with exception for BPD where estimates were similar at 5.5% and 5.1%, respectively (Table 2).

### 4.3 Cumulative lifetime risk, age of onset distributions, & cohort effects

Projections of estimated future risk as of age 65 years for fixed percentiles of the AOO distributions are presented in Table 3. The median AOO of any anxiety or mood disorder was 26 years with considerable variation among the individual disorders (Table 3). The IQR for BPD was the narrowest (18–31), which means that 25% of those who will ever develop BPD will have it by age 18, while 75% of them will have it by age of 31. In contrast, the IQR for GAD was the widest ranging between 24 and 49 (Table 3).

**TABLE 3:** Projected lifetime risk at age 65, ages at selected probabilities on the standardized age of onset distributions, and cohort (age at interview) as a predictor of lifetime risk of DSM‐5/CIDI disorders in Qatar.

Disorder category	Projected LT risk age 65^b^		Ages at selected age of onset percentiles^b^								Younger cohorts (age at interview) compared to respondents aged 50+^c^						Test^c^
											18–29		30–39		40–49		Statistics
	%	SE	5	10	25	50	75	90	95	99	OR	95% CI	OR	95% CI	OR	95% CI	$\chi_{3}^{2}$ (*p*)
Panic disorder	4.6	0.7	17	19	24	37	47	59	59	59	23.6*	11.0–50.5	9.3*	4.8–18.1	2.9*	1.6–5.0	23.9 (<0.001)*
Generalized anxiety disorder	9.8	1.0	17	19	24	35	49	59	59	61	14.8*	9.2–23.9	5.6*	3.3–9.5	2.3*	1.6–3.5	51.8 (<0.001)*
PCL‐SC PTSD^a^	24.7	1.4	12	16	20	26	37	47	56	60	7.1*	4.7–10.6	3.3*	2.3–4.6	2.0*	1.4–2.7	33.3 (<0.001)*
Obsessive‐compulsive disorder^a^	4.4	0.6	12	16	20	28	41	51	53	60	6.3*	2.8–13.9	4.7*	2.2–10.2	1.5	0.6–3.4	10.2 (<0.001)*
First/Any anxiety disorder^a^	28.6	1.3	11	16	20	26	37	47	56	59	6.7*	4.8–9.6	3.3*	2.4–4.5	2.0*	1.5–2.6	40.3 (<0.001)*
Major depressive disorder	17.4	1.0	17	19	23	31	39	51	56	59	20.5*	14.4–29.1	5.7*	4.2–7.6	2.7*	1.9–4.0	122.0 (<0.001)*
Bipolar board disorder	6.4	0.5	13	16	18	24	31	41	51	60	4.9*	3.1–7.8	3.4*	2.2–5.1	2.2*	1.5–3.4	17.1 (<0.001)*
First/Any mood disorder	23.4	0.9	16	17	21	28	37	47	55	59	13.1*	9.8–17.5	4.9*	3.8–6.4	2.6*	2.0–3.4	125.6 (<0.001)*
First/Any disorder^a^	36.4	1.4	12	16	19	26	36	47	56	59	7.1*	5.3–9.6	3.3*	2.5–4.3	2.0*	1.5–2.5	62.5 (<0.001)*

Abbreviation: LT, Lifetime.

^a^ Disorder was only assessed in the Part 2 sample (*n* = 2583).

^b^ Projected lifetime risk and ages at selected probabilities of onset results reflect weighted data, with 50 strata and 100 clusters.

^c^ Cohort or age at interview results reflect weighted person‐year level data. Models include time intervals as controls.

*Significant age difference at the 0.05 level.

The projected lifetime risk of any mood or anxiety disorder was 36.4%, which was about 8% higher than the lifetime prevalence of any disorder at 28.2% reported in Table 2.

Consistent with lifetime prevalence of any anxiety disorder (21.5%) being higher than any mood disorder (17.3%), the projected lifetime risk was also higher for anxiety disorders (28.6%) compared to mood disorders (23.4%). Comparing the lifetime risk with lifetime prevalence in absolute terms, the percentage increase for any mood and any anxiety disorders were similar, approximately 6% and 7%, respectively.

Similar to individual disorders with the highest prevalence (Table 2), PTSD had the highest lifetime risk at 24.7% followed by MDD at 17.4% (Table 3). However, the relative increase in the most common disorders for lifetime risk compared to lifetime prevalence was higher for MDD at 45% than PTSD at 36%.

This difference between projected lifetime risk and prevalence are partly due to the variability in the AOO across disorders with larger increases due to later AOO distributions. As shown in Table 3, mood disorders had a typically later median AOO (28 years) compared to the median AOO for anxiety disorders (26 years), with largest differences found at the specific disorder level. For example, the median AOO was earliest in BPD at 24 years and latest in PD at 37 years (Table 3). This explains why PD exhibited the largest relative increase at 84%, while BPD exhibited the smallest relative increase in projected risk compared to prevalence at 21%.

Cohort effects are also evident in the projected lifetime risk for mental disorders with the youngest (18–29 years) at the time of interview showing the highest odd ratios (ORs range of 4.9–23.6) for all six disorders relative to oldest (50+) age cohorts (Table 3). The ORs for other age‐at‐interview categorical comparisons (30–39 and 40–49) were also elevated relative to the oldest cohort (ORs range from 2 to 9) for all disorders with the exception of the OCD lifetime risk comparison of the 40–49 years relative to 50+ years participants (OR = 1.5, 95% CI: 0.6–3.4; Table 3).

Results from the sensitivity analysis investigating the validity of the assumption of constant differences in these observed cohort effects across the full AOO range are shown in Appendix Table A1. These results replicate the general trend of more recent cohorts having increased lifetime risk of any DSM‐5 mental disorder across early‐, middle‐, and late‐onset subsamples of our data.

### 4.4 Sociodemographic correlates of lifetime risk

Table 4 shows sociodemographic differences in lifetime risk of any anxiety, any mood, or any of these two broad disorder categories. Women in Qatar's Arab population had a higher lifetime risk of any anxiety or mood disorders compared to men (OR = 1.9, 95% CI = 1.6–2.2).

**TABLE 4:** Sociodemographic correlates of lifetime risk of DSM‐5 disorders in the World Mental Health Qatar (WMHQ) study.

Predictor category	Predictor variable	Any anxiety disorder^a^		Any mood disorder		Any mood/Anxiety disorder^a^	
		OR	95% CI	OR	95% CI	OR	95% CI
Gender	Female	1.9*	(1.6–2.3)	1.9*	(1.6–2.2)	1.9*	(1.6–2.2)
	Male	1.0	‐‐	1.0	‐‐	1.0	‐‐
	**Test statistics**	$\chi_{1}^{2}$ (*p*)	53.2 (<0.001)*	$\chi_{1}^{2}$ (*p*)	80.3 (<0.001)*	$\chi_{1}^{2}$ (*p*)	74.7 (<0.001)*
Time‐varying education	Student	0.5*	(0.4–0.7)	0.7*	(0.5–0.9)	0.6*	(0.5–0.7)
	Low	0.6*	(0.4–0.9)	0.5*	(0.4–0.7)	0.6*	(0.4–0.8)
	Low average	1.0	(0.8–1.3)	1.0	(0.8–1.2)	1.0	(0.8–1.2)
	High average	0.7*	(0.5–1.0)	1.0	(0.8–1.3)	0.8	(0.6–1.0)
	High	1.0	‐‐	1.0	‐‐	1.0	‐‐
	**Test statistics**	$\chi_{4}^{2}$ (*p*)	7.8 (<0.001)*	$\chi_{4}^{2}$ (*p*)	5.2 (0.001)*	$\chi_{4}^{2}$ (*p*)	7.5 (<0.001)*
Time‐varying ever marriage	Ever married	0.9	(0.7–1.2)	0.9	(0.7–1.0)	0.9	(0.7–1.1)
	Never married	1.0	‐‐	1.0	‐‐	1.0	‐‐
	**Test statistics**	$\chi_{1}^{2}$ (*p*)	0.6 (0.450)	$\chi_{1}^{2}$ (*p*)	2.4 (0.128)	$\chi_{1}^{2}$ (*p*)	1.1 (0.299)
Time‐varying migration	Migrated	1.3*	(1.1–1.7)	1.5*	(1.2–1.7)	1.3*	(1.1–1.6)
	Never migrated	1.0	‐‐	1.0	‐‐	1.0	‐‐
	**Test statistics**	$\chi_{1}^{2}$ (*p*)	7.8 (0.008)*	$\chi_{1}^{2}$ (*p*)	19.1 (<0.001)*	$\chi_{1}^{2}$ (*p*)	11.9 (0.001)*

*Note*: Results reflect weighted person‐year level data. Models include time intervals and data stacks (if it is a sub‐group model) as controls. To avoid prediction of the first onset of any individual mental disorder within each sub‐group, the analysis sample for each sub‐group was created by stacking the person‐year sample for each mental disorder within each sub‐group on top of each other (see Table 1), for a total of 4 data stacks measuring any anxiety, 2 data stacks for any mood, and 6 data stacks measuring any dx. Dummy variables for n‐1 stacks were included as controls in each sub‐group model. For Time‐varying education, Low refers to those who have Preparatory School or less as their highest education; Low Average ‐ Secondary School; High Average ‐ Diploma; High ‐ Bachelor's and more.

^a^ Disorder was only assessed in the Part 2 sample (*n* = 2583).

*Significant difference at the 0.05 level.

Compared with those with lower education, those with higher education (Bachelor's degree or more) had a greater lifetime risk of anxiety or mood disorder ($\chi_{4}^{2}$ = 7.5, *p* < 0.001). Migrants relative to non‐migrants were at higher lifetime risk of any anxiety or any mood disorder (OR = 1.3, 95% CI = 1.1–1.6). Marital status was not significantly associated with lifetime risk of any anxiety or mood disorders ($\chi_{1}^{2}$ = 1.1, *p = 0*.*299*). The disorder‐specific sociodemographic differences in lifetime risk for individual mood (Appendix Table A2) and anxiety disorders (Appendix Table A3) are presented as supplementary material.

### 4.5 Lifetime treatment across healthcare and non‐healthcare sectors

Among participants with any lifetime anxiety or mood disorder who received treatment, 51.3% received treatment from SA, 50.4% received treatment in the MHS, 25.0% received treatment in the GMP, 15.8% received treatment in the CAM sectors, and 10.8% were hospitalized (Table 5). As shown in Table 5, the distribution of treatment in our sample across healthcare and non‐healthcare sectors did not vary much by disorder class (mood vs. anxiety). Overall, 63.5% received treatment in the healthcare and 59.9% in the non‐healthcare sectors.

**TABLE 5:** Proportional lifetime treatment across treatment sectors among respondents who obtained lifetime treatment.

Disorder	Healthcare treatment								Non‐healthcare treatment						Any treatment		Number of unweighted respondents
	Mental health hospitalization		Mental health specialty		General medical professional		Any healthcare		Spiritual advisor		CAM		Any non‐healthcare				
	%	SE	%	SE	%	SE	%	SE	%	SE	%	SE	%	SE	%	SE	
Panic disorder	19.6	5.0	62.1	5.8	29.3	5.7	74.5	5.5	52.9	6.2	21.2	5.3	64.8	5.8	100.0	0.0	66
Generalized anxiety disorder	17.4	3.7	64.4	4.7	28.4	4.6	79.1	4.1	45.9	5.0	16.7	3.4	54.3	4.5	100.0	0.0	103
PCL‐SC PTSD	11.4	2.0	51.0	2.8	26.4	2.9	63.8	2.8	50.8	3.1	18.2	2.6	60.1	3.0	100.0	0.0	282
Obsessive‐compulsive disorder	15.1	4.8	57.8	6.4	30.0	5.2	68.8	6.6	55.7	6.8	14.8	5.1	66.0	6.2	100.0	0.0	67
Any anxiety	11.2	1.8	52.1	2.6	26.4	2.6	65.5	2.6	49.0	2.8	18.3	2.4	59.0	2.6	100.0	0.0	331
Major depressive	11.0	2.2	55.8	3.8	22.8	2.8	68.4	3.8	50.8	3.7	11.4	2.4	56.6	3.7	100.0	0.0	202
Broad bipolar disorder	16.7	3.9	45.5	6.2	24.8	3.8	58.6	5.8	60.8	5.3	13.9	3.8	67.3	4.6	100.0	0.0	82
Any mood	12.7	2.0	52.7	3.2	23.4	2.1	65.5	3.0	53.8	3.0	12.1	1.8	59.8	2.7	100.0	0.0	284
Any disorder	10.8	1.5	50.4	2.4	25.0	2.2	63.5	2.2	51.3	2.5	15.8	2.0	59.9	2.2	100.0	0.0	402
1 anxiety disorder	6.1	1.6	48.0	3.6	24.7	3.8	60.6	3.7	44.5	4.0	20.4	3.5	56.4	3.9	100.0	0.0	196
2 anxiety disorders	20.3	5.1	53.9	5.2	26.4	5.0	72.2	6.1	57.3	4.6	12.0	4.0	62.3	4.9	100.0	0.0	92
3 anxiety disorders	15.8	6.2	61.4	8.3	38.6	7.5	70.4	7.6	60.1	8.9	21.2	6.7	68.1	7.2	100.0	0.0	34
4 anxiety disorders	23.9	14.7	92.7	7.2	20.5	13.2	92.7	7.2	29.6	15.1	22.4	14.3	52.0	17.1	100.0	0.0	9
2 or more anxiety	19.4	3.6	58.6	4.5	29.0	4.1	73.2	5.0	56.0	4.3	15.1	3.2	63.1	3.8	100.0	0.0	135
3 or more anxiety	17.6	6.1	68.3	7.2	34.6	5.7	75.3	6.7	53.4	8.1	21.4	6.2	64.6	7.4	100.0	0.0	43
1 disorder	7.2	2.1	41.7	4.2	25.0	3.9	54.7	4.0	51.1	4.6	16.6	3.6	60.6	3.8	100.0	0.0	166
2 disorders	6.2	2.7	56.5	3.8	20.2	3.4	68.4	4.1	46.8	4.4	15.8	2.9	56.9	4.2	100.0	0.0	120
3 disorders	23.1	6.1	54.6	5.8	26.6	4.9	72.6	6.3	57.4	4.8	9.7	4.3	59.4	4.8	100.0	0.0	77
4 disorders	15.7	6.6	56.6	9.3	40.8	8.2	66.7	8.6	61.1	9.6	23.8	7.7	70.1	7.5	100.0	0.0	30
5 disorders	23.9	14.7	92.7	7.2	20.5	13.2	92.7	7.2	29.6	15.1	22.4	14.3	52.0	17.1	100.0	0.0	9
2 or more disorders	13.7	2.5	57.4	3.0	25.0	2.4	70.5	3.2	51.4	3.1	15.1	2.2	59.3	2.8	100.0	0.0	236
3 or more disorders	21.2	4.1	58.3	4.9	29.8	4.1	72.7	5.3	56.0	4.6	14.5	3.6	61.6	3.6	100.0	0.0	116
4 or more disorders	17.7	6.5	65.3	7.9	35.9	6.1	73.0	7.4	53.6	8.6	23.5	6.9	65.8	7.7	100.0	0.0	39

However, the pattern of equal proportional treatment across healthcare and non‐healthcare sectors was not observed for specific disorders with: (1) GAD with 25% higher treatment in healthcare (79.1%) versus non‐healthcare (54.3%) sectors; (2) MDD with 12% higher treatment in healthcare (68.4%) versus non‐heatlhcare (56.6%); (3) PD with roughly 10% higher treatment in healthcare (74.5%) versus non‐healthcare (64.8%); (4) BPD with 9% treatment lower in healthcare (58.6%) versus non‐healthcare (67.3%); (5) PTSD with 4% higher treatment in healthcare (63.8%) versus non‐healthcare (60.1%); and (6) OCD with 3% higher treatment in healthcare (68.8%) versus non‐healthcare (66.0%).

Within the healthcare sector, the three disorders that received the highest treatment from MHS were (Table 5): GAD (64.4%), PD (62.1%), and OCD (68.8%). These same disorders also received the highest rates of treatment from GMP (OCD: 30.0% and PD: 29.3%, GAD: 28.4%) and were two of the top contributors to mental health hospitalization (PD: 19.6%, GAD: 17.4%, and BPD 16.7%).

Within the non‐healthcare sector, treatment is more common by SA than CAM sectors across all six disorders (Table 5). The three disorders that received the highest treatment from spiritual advisors were: PD (52.9%), OCD (55.7%) and BPD (60.8%). As shown in Table 5, both PD and PTSD were the top two disorders who received the highest CAM at 21.2% and 18.2%, respectively, followed by GAD (16.7%) and OCD (14.8%).

### 4.6 Lifetime treatment, initial contact and duration of delay

Treatment contact in the year of disorder onset was low for any disorder at only 13.5% (Table 6) and was higher for any mood (20.4%) than any anxiety disorder (15.7%). The treatment contact increased overall to 30.8% for any disorder at the time of the interview and the treatment contact proportions were equal for any mood (34.4%) and for any anxiety (33.4%) disorders to date (Table 6). Additionally, the majority (68.4%) of the people with any lifetime disorder eventually sought treatment if disorder persisted 50 years from onset with higher projected lifetime treatment for any mood (80.1%) compared to any anxiety disorder (68.1%).

**TABLE 6:** Proportional treatment contact in the year of disorder onset and median duration of delay among cases that subsequently made treatment contact.

	% making treatment contact in year of onset	% making contact by this interview	% making treatment contact by 50 years from first onset	Median delay	Delay IQR, P25	Delay IQR, P75	*N*
Panic disorder	44.9	56.5	79.6	5	2	10	119
Generalized anxiety disorder	21.9	37.3	69.3	4	2	10	268
PCL‐SC PTSD	17.6	33.8	71.8	6	2	14	724
Obsessive‐compulsive disorder	26.1	49.5	81.3	5	3	11	133
Any anxiety disorders	15.7	33.4	68.1	5	2	14	876
Major depressive disorder	21.6	34.7	72.1	5	3	9	560
Broad bipolar disorder	17.7	33.7	84.1	8	3	13	234
Any mood disorders	20.4	34.4	80.1	6	3	11	794
Any disorder	13.5	30.8	68.4	5	2	13	1177
1 anxiety disorder	11.8	27.7	63.1	5	2	15	605
2 anxiety disorders	17.6	47.7	65.1	6	3	12	190
3 anxiety disorders	13.0	51.6	78.0	8	5	15	65
4 anxiety disorders	11.8	58.7	79.1	5	3	17	16
2 or more anxiety disorders	16.1	49.3	71.2	7	3	13	271
3 or more anxiety disorders	12.8	53.0	79.6	8	4	15	81
1 disorder	12.7	23.2	53.7	5	2	12	626
2 disorders	13.0	35.2	90.1	5	3	10	326
3 disorders	17.7	48.2	65.9	7	3	13	155
4 disorders	13.9	57.0	86.4	8	4	14	55
5 disorders	12.5	61.7	89.9	5	3	17	15
2 or more disorders	14.4	41.7	87.1	6	3	13	551
3 or more disorders	16.4	51.2	77.2	7	3	13	225
4 or more disorders	13.6	58.0	87.9	8	3	15	70

The highest treatment proportions in the year of onset was for PD at 44.9% followed by OCD at 26%, GAD or MDD at roughly 22%, and BPD or PTSD at 18% (Table 6).

The median delay in receiving treatment for any anxiety or mood disorder was 5 years with IQR of 2–13 (Table 6). The median delay for any anxiety disorder was 5 years (IQR = 2–14) and for any mood disorder was 6 years (IQR = 3–11). Increased number of disorders appeared to prolong median duration of delay from 6 years (IQR = 3–13) for those with two or more disorders to 8 years for those with four or more disorders (IQR = 3–15). Among the specific disorders, GAD had the shortest and BPD had the longest median durations of delay among cases that subsequently made treatment contact at 4 years (IQR = 2–10) and 8 years (IQR = 3–11), respectively (Table 6).

**FIGURE 1:**
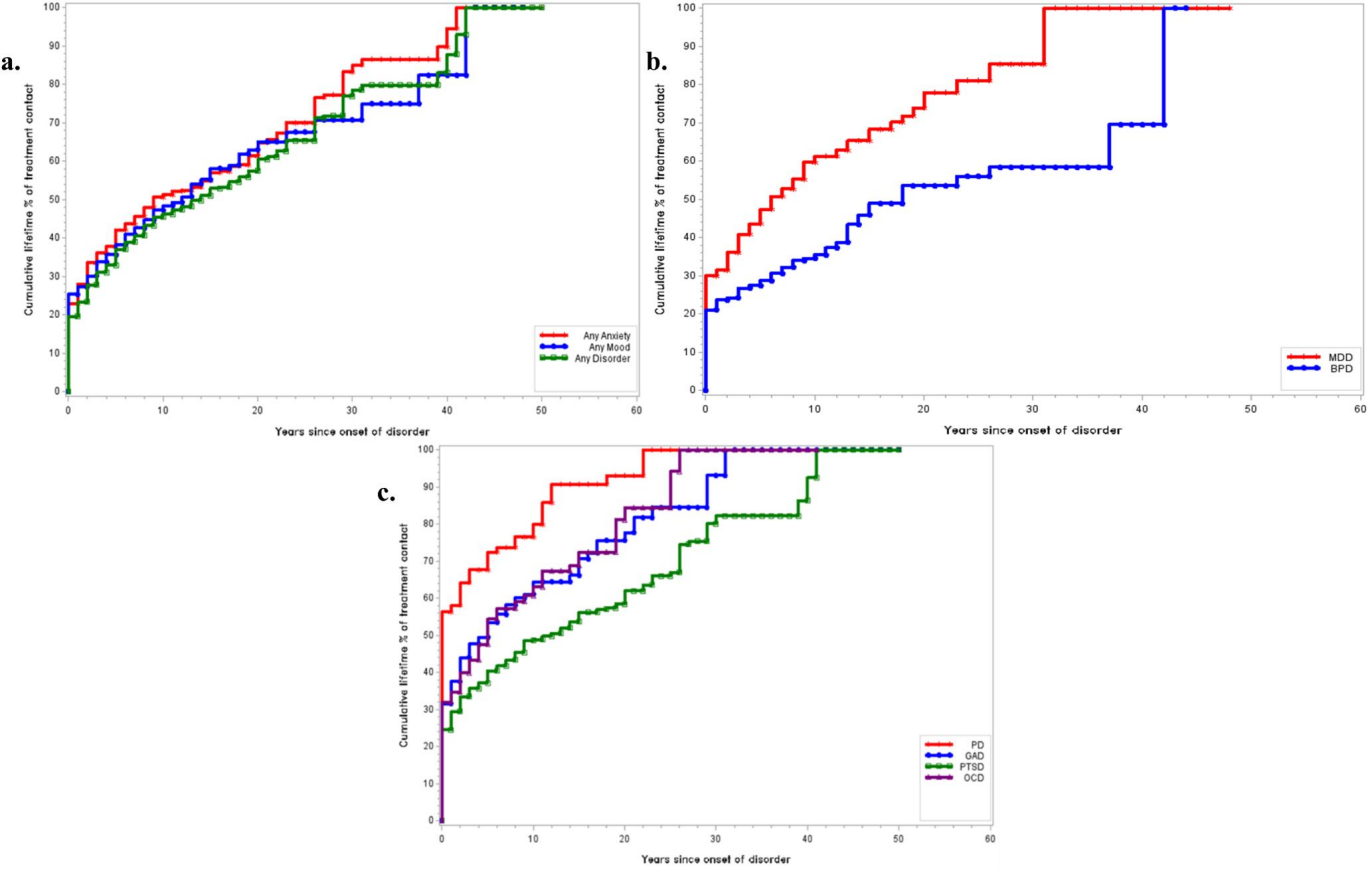
(a) Cumulative lifetime % of treatment contact for any anxiety or mood disorders from year of onset. (b) Cumulative lifetime % of treatment contact for mood disorders from year of onset. (c) Cumulative lifetime % of treatment contact for different anxiety disorders from year of onset.

Survival curves for the cumulative lifetime probabilities of treatment contact are also shown in Figure 1a for any disorder, Figure 1b for specific mood and in Figure 1c for anxiety disorders. The proportions for eventually receiving treatment after 50 years from onset are higher for BPD (84.1%) than MDD (72.1%). The projected proportions for treatment contact also varied across the different anxiety disorders with OCD (81.3%) and PD (79.6%) among the highest, while PTSD (71.3%) and GAD (69.3%) among the lowest to receive treatment for those whose disorders persist 50 years.

### 4.7 Predictors of lifetime treatment

The only predictors of lifetime treatment were cohort and AOO. For both anxiety and mood disorders, the pattern of higher odds of treatment in more recent cohort (relative to oldest cohort) and later AOO (relative to early onset) were consistently associated with higher odds of treatment. In contrast, gender, education, marital status, and migrant status did not predict lifetime treatment for any anxiety or mood disorder in Qatar's Arab population (Table 7).

**TABLE 7:** Socio‐demographic predictors of lifetime treatment contact for any disorder.

Predictor	Any anxiety disorder			Any mood disorder				Any disorder			
	OR	95% CI	*p*‐value	Odds ratio	95% CI		*p*‐value	OR	95% CI		*p*‐value
Age at interview											
18–29	3.0	1.7–5.4		3.4	1.4	8.5		3.5	1.9	6.3	
30–39	1.8	1.1–2.9		2.1	0.9	4.8		1.9	1.1	3.4	
40–49	1.2	0.7–2.0		1.7	0.9	3.4		1.4	0.8	2.4	
$\chi_{3}^{2}$	6.64*		0.001	2.89*			0.045	8.62*			0.000
Age at onset	1.4	1.1–1.7		1.6	1.2	2.1		1.4	1.2	1.8	
$\chi_{1}^{2}$	10.95*		0.002	9.98*			0.003	13.09*			0.001
Gender											
Female	1.1	0.9–1.4		1.0	0.8	1.4		1.1	0.9	1.4	
$\chi_{1}^{2}$	0.64		0.428	0.06			0.808	0.94			0.338
Education											
Student	0.7	0.5–1.1		0.8	0.5	1.4		0.8	0.5	1.2	
Low	0.8	0.5–1.3		0.8	0.4	1.4		0.7	0.4	1.2	
Low‐average	1.1	0.8–1.5		1.1	0.8	1.6		1.1	0.8	1.5	
High‐average	0.8	0.4–1.6		0.7	0.4	1.2		0.9	0.5	1.7	
$\chi_{4}^{2}$	1.5		0.216	1.6			0.187	1.76			0.152
Marital status											
Ever married	1.1	0.7–1.6		1.1	0.7	1.6		1.1	0.7	1.5	
$\chi_{1}^{2}$	0.11		0.743	0.22			0.638	0.12			0.73
Migration status											
Migrated	0.9	0.7–1.3		0.9	0.7	1.3		0.9	0.7	1.2	
$\chi_{1}^{2}$	0.32		0.574	0.23			0.633	0.62			0.437

*Note*: Based on a person‐year survival analysis controlling for AOO, time since onset, age at interview, sex, time‐varying education, time‐varying marital status, and time‐varying migration status, All treatment models used Part II weights. Analysis restricted to the subset responding to the long interview (*n* = 2583).Respondents who said they did talk to a medical doctor or other professional AND did not report their age of first treatment were excluded from the LT treatment analyses (*n* = 8).

*p < 0.05.

## 5 DISCUSSION

The WMHQ is the first national mental health survey conducted to date. During that time, the global COVID‐19 pandemic was resolving in many, if not, most parts of the world. To date, our study is the first to provide data on the prevalence and the sociodemographic correlates of common mental disorders and their treatment seeking in the general population of Qatar. Our study also provides current data to gauge the progress of Qatar's mental health service toward community‐based care in the past few decades, a period that also encompassed the COVID‐19 world pandemic.

### 5.1 Lifetime prevalence & sociodemographic correlates of mood & anxiety disorders

Our current data show that the lifetime prevalence of any mood or anxiety disorder in Qatar was 28%. There was slightly higher prevalence of any lifetime anxiety disorder than any mood disorder (21.5% vs. 17.3%). However, there was variable prevalence among the disorders assessed. The most prevalent was PTSD, followed by MDD, GAD, BPD, OCD, and lastly PD.

We unexpectedly found a preponderance of trauma‐driven anxiety as in PTSD over other anxiety‐related disorders such as GAD, OCD and PD. This finding raises the question as to why, in a peaceful country like Qatar, there should be a high prevalence of lifetime PTSD (18.2%) compared to the US (6.8%) (Kessler et al., 2005) and other Middle East countries such as Iraq (2.5%) (Alhasnawi et al., 2009), Saudi (3.3%) (Altwaijri et al., 2020), and Lebanon (3.4%) (Karam et al., 2008). There are many types of stressful events that can lead to PTSD shared across countries for example, accidents, abuse etc. On top of these, it is possible that non‐Qatari Arab residents in Qatar, many of whom are work migrants from conflict‐afflicted neighboring Arab countries including Lebanon, Syria, and Iraq, face additional stress (Khaled & Gray, 2019). In fact, 22% of those who never migrated in our sample were non‐Qatari Arabs who were born in Qatar. For non‐Qatari citizens born in Qatar, the struggle for national identity is a complex issue; this portion of society do not belong to one particular nation as many other expatriates do, but neither do they fit into mainstream Qatari society. Being residents, without citizenship rights under Qatari law, may contribute to their social vulnerability. Rights and privileges of Qatari citizenship is largely limited to offspring born to male Qatari citizens.

In the WMHQ study, the focus was mainly on anxiety and mood disorders, so direct and appropriate comparisons can be made in prevalence of these disorders between Qatar and other countries. Here, Qatar, alongside the US, had one of the highest lifetime prevalence of GAD of any country at 5.7%. Lifetime prevalence of PD in Qatar of 2.5% was equivalent to that in Saudi Arabia (2.3%), lower than in the US (4.7%) but still higher than in Iraq (1.4%) and in Lebanon (0.5%). In contrast, although Qatar had the lower OCD rates than other Arab countries in the WMH Surveys (at 2.9%, vs. 4.2% in Saudi Arabia and 4.6% in Iraq), it was still higher than the rate of OCD in the US (1.6%). It is possible that religious routines like ablution prior to prayer five times a day practiced by Muslims can evolve in some people into broader obsessional behaviors. Future studies should investigate the determinants of OCD in Qatar and other Arab countries.

The lifetime prevalence of any anxiety disorder in our study (21.5%) was at the higher end along with that in the US (28.8%) and Saudi Arabia (23.2%) (Altwaijri et al., 2020; Kessler et al., 2005), both highly affluent countries but different culturally. However, anxiety prevalence was higher in Qatar than in other Middle East countries that share cultural norms such as Lebanon (16.7%) and Iraq (13.8%), but in contrast to Qatar, have faced recent unrest (Alhasnawi et al., 2009; Karam et al., 2008). Naturally, anxiety has many potential causes unrelated to affluence or societal pressures so broad conclusions cannot be made at this stage. However, these differences do underpin the importance of identifying further the specific local contributing factors in those countries. Of note, these studies were conducted few decades ago leaving the possibility that the lifetime prevalence of these disorders in both countries may have risen in the younger cohorts since then.

The lifetime prevalence of any mood disorder was highest in the US at 20.8% (Kessler et al., 2005), followed by Qatar (17.3%). These rates were marginally higher than those in Lebanon (12.6%) but much higher than those in Saudi Arabia (9.3%) and Iraq (7.5%) (Alhasnawi et al., 2009; Altwaijri et al., 2020; Karam et al., 2008). The higher rate of mood disorders in Qatar than other Middle East countries may reflect the inclusion of migrants and non‐naturalized citizens in the Qatar WMHQ study. These groups were typically excluded from the single nationality samples of the studies conducted in other Arab countries within the WMH consortium. A previous population‐based survey showed that migrants in Qatar have higher rates of depression as assessed by an ultra‐brief depression‐screening tool and negative perceived quality of life in Qatar was strongly associated with depressive symptomology among migrants of Qatar (Khaled & Gray, 2019).

### 5.2 Cumulative lifetime risk, age of onset, & cohort effects

The projected lifetime risk of any mood or anxiety disorder in Qatar was roughly 36% (i.e. of those who live to 65 years in our sample, roughly 36% of them are predicted to have at least one mood or anxiety disorder). This risk was higher for anxiety compared to mood disorders (29% vs. 23%). Mood disorders had a typically later median AOO (28 years) compared to the median AOO for anxiety disorders (26 years). Consistent with lifetime prevalence data, individual disorders with the highest lifetime prevalence also had highest lifetime risk. In our study, PTSD had the highest lifetime risk (25%) followed by MDD (17%).

Projections of estimated future risk as of age 65 years were also computed for fixed percentiles of the AOO distributions in Qatar. The median AOO of any anxiety or mood disorder in Qatar of 26 years (IQR = 19–36) was much later than the median AOO in Saudi Arabia at 13 years (IQR = 8–19) (Altwaijri et al., 2020), the US at 14 years (IQR = 7–24) (Kessler et al., 2005), and 19 years (IQR = 11–35) in Lebanon (Karam et al., 2008), but earlier than that in Iraq at 29 years (IQR = 14–54) (Alhasnawi et al., 2009). Higher domestic violence rates in both US (“National Statistics,” 2019) and Saudi Arabia (Human Rights Council, 2009) may contribute to the earlier AOO distribution compared to countries with lower rates of domestic violence like Qatar. Additionally, the remarkably later AOO distribution for war‐torn countries like Lebanon and Iraq compared to Saudi and the US highlight differences in war‐related compared to domestic‐related violence with the latter more likely to target children and underage dependents (Childhood Domestic Violence Association, 2014). These findings may support the argument that domestic‐related may be as harmful as war‐related violence in increasing the burden of anxiety‐related disorders through earlier AOO. As information about lifetime occurrence and age of first occurrence of trauma exposures of many types was obtained in the survey, these speculations can be investigated in future analyses of the data.

In our sample, the observation that ORs decreased with age is consistent with the possible existence of cohort effects. Similar patterns have been found in other WMH surveys (Table 3). The implications is that the lifetime prevalence of any mood or anxiety disorder in Qatar is greater in the younger cohorts. One explanation could be that, alongside Qatar's rapid economic development and urbanization and opening up to outside events, such as hosting of the 2022 FIFA World Cup, an enhanced awareness of mental health, increased mental health literacy, and reduced stigma could facilitate younger people to report mental illness. The implication for Qatar's mental health service is that younger cohorts are increasingly likely to report and seek treatment for their mental health compared to older cohorts who may view having a mental illness as a weakness of personality or punishment by God (Dardas & Simmons, 2015; Jurewicz, 2015; Rickwood et al., 2007).

### 5.3 Sociodemographic correlates of lifetime risk

When compared to those who always resided in the country, migrants of Qatar had significantly higher lifetime risk of mood or anxiety disorder than non‐migrants. There are no comparative data for the other Arab countries in the WMH consortium as none of these studies included multinational population samples. As far as we know, this is one of the largest multinational studies conducted in the Middle East to date that shed light on the vulnerability of migrants in Qatar.

In Qatar, women also significantly differed from men in their lifetime risk of any mood or any anxiety disorder. These findings were consistent with most other studies to date including findings from other Arab‐speaking populations within the WMH consortium like Saudi Arabia (Altwaijri et al., 2020) and Lebanon (Karam et al., 2008). In relation to specific disorders, these gender differences persisted for all disorders assessed in the study with exception of BPD. Most other studies, but not all, report an equal risk of BPD between men and women (Diflorio & Jones, 2010). Findings from our study also shed light on the importance of supporting mental health prevention, screening, and early treatment initiatives for women in Qatar. This builds on the progress that Qatar already made so far in developing women's mental health program providing peri‐ and post‐natal specialized services (Alabdulla et al., 2022).

Another important finding is that the lifetime risk of students and participants with low education (preparatory school or less) were significantly lower compared to participants with highest level of education (Bachelor's degree or higher). Otherwise, all other education categories were similar in risk to those with the highest education level category. The beneficial effects of being a student or someone with low education in Qatar may reflect the benefits from government initiatives, scholarships, and social programs that focus on those with low‐income including students and those with special needs.

### 5.4 Lifetime treatment, initial contact, and duration of delay

In Qatar, less than 1 in 3 (30.8%) participants sought treatment by the time of the interview for any lifetime disorder. Similar treatment proportions were reported for any anxiety (33.4%) or mood (34.4%) disorder. These estimates are comparable to overall lifetime treatment estimates reported for Saudi Arabia (28.3%) including rates for any anxiety (33.0%) or mood disorder (35.4%) (Al‐Subaie et al., 2020). Overall, these rates were generally lower than treatment rates reported for any lifetime anxiety (37.3%) or mood (49.2%) disorder for war‐afflicted Lebanon (Karam et al., 2019) and much lower than lifetime treatment rates reported in high‐income countries (Wang et al., 2007).

Notably, treatment contact proportions in the year of disorder onset were low (13.5%) in Qatar. This is consistent with findings from most WMH surveys where there was substantial delay in seeking treatment for most disorders (Wang et al., 2007). In Qatar, the proportion of treatment in the year of onset was also low, but higher for any mood (20.4%) than any anxiety disorder (15.7%). These findings are supported by the wider literature showing inadequate recognition and treatment for patients with anxiety compared to other psychiatric disorders including depression in primary care, where most treatment contacts are typically received (Gelenberg, 2000). Notably, among the anxiety disorders—our findings support highest treatment in the year of onset for PD (45%), followed by GAD (22%), and lowest for PTSD (18%). This pattern is consistent with Qatar's context of high mental illness stigma (Zolezzi et al., 2017) and may explain why people were more likely to seek treatment for somatic‐rather than generalized‐ or trauma‐based anxiety.

The median delay in receiving treatment was 5 years (IQR = 2–13) with 75% of people with any lifetime disorder in Qatar experiencing treatment delays for up to 13 years.

Higher delays were observed for any anxiety (14 years) than any mood disorder (11 years). Among the specific disorders, GAD had the shortest median (4 years) duration of treatment delay compared to BPD with the longest median duration of delay among cases that subsequently made treatment contact (8 years). Most studies conducted in the Middle East found pervasive treatment delays for the majority of mental disorders, consistent with or higher than those reported in our study. For example, the median treatment delay for any lifetime disorder reported in Saudi Arabia was 8 years with IQR of 3–15 years (Al‐Subaie et al., 2020). The proportions for making contact in the first year of onset in Saudi Arabia were much lower compared to Qatar (4.1% vs. 13.5%) and by the time of the interview (24.4% vs. 30.8%) (Al‐Subaie et al., 2020). These higher delays in treatment contacts in Saudi Arabia compared to Qatar were also observed for any anxiety and any mood disorder (Al‐Subaie et al., 2020). On the other hand, these treatment delays were comparatively lower in high‐income countries than in Qatar. For example, in the US, the range for treatment delays for any mood disorder was 6–8 years compared to a range of 9–23 years for any anxiety disorder (Wang et al., 2005).

Our survival‐based projections of the proportions of cases eventually receiving treatment after 50 years from onset showed that the majority (68.4%) of the people with any lifetime disorder in Qatar would eventually seek treatment. Consistent with previously published studies to date, our data also show that eventually the majority of people with any anxiety or mood disorder make a treatment contact. Here, there is a higher projected lifetime treatment for any mood (80.1%) compared to any anxiety disorder (68.1%). Among the specific disorders, BPD (84.1%) was among the highest followed by OCD (81.3%), PD (79.6%), MDD (72.1%), PTSD (71.3%), while GAD (69.3%) was the category with the lowest proportion eventually receiving treatment. Similar findings were also reported in the US and other countries highlighting the importance of focusing on timely and disorder specific interventions to reduce treatment delays and promote prompt help seeking to ameliorate the burden of unmet need in the treatment of mental disorder in the population.

### 5.5 Lifetime treatment across healthcare and non‐healthcare sectors

The lifetime treatment estimates among those with a lifetime disorder were overall similar across healthcare compared to non‐healthcare in Qatar. The differences in utilization observed between the two sector types for specific disorders were even more similar for conditions like OCD and PTSD compared to GAD that require more long‐term, ongoing or more‐intensive specialized mental health treatment (e.g. psychotherapy). Previously published studies have demonstrated high stigma and negative attitudes toward effectiveness of treatment for mental disorders in Qatar (Zolezzi et al., 2017) and Arab culture (Ghuloum et al., 2010; Zolezzi et al., 2017). This may also explain why some of the conditions that had the highest proportion of lifetime hospitalization‐related treatment in our study (e.g. PD and BPD) were also conditions that received largest lifetime treatment provided by the SA (BPD and PD) and CAM (PD). As service utilization in the community is largely influenced by cultural beliefs, literacy levels, and stigma, future studies need to explore how interventions targeting these factors may improve access to the mental health service in Qatar's general population.

Our findings show that multimorbidity may be an important contributor to disproportional treatments between the sectors in Qatar with the largest discrepancy in treatment proportions observed in healthcare (92.7%) versus non‐healthcare (52.0%) sectors for those with five disorders. This is consistent with existing literature showing that this phenomenon is common in psychiatry and is typically associated with high healthcare utilization requiring more integrated care (Langan et al., 2013). This finding is of importance for Qatar's developing mental health service as more integrated care pathways with primary health care centers across the country are needed to ensure continuous and consistent care, particularly for patients with multimorbidity whose needs are often complex (Langan et al., 2013).

### 5.6 Predictors of lifetime treatment

Like most WMH studies, we found that lifetime treatment in Qatar was significantly associated with younger cohort and older rather than younger AOO (Wang et al., 2007). Unlike previous studies, we did not find any association between odds of treatment in Qatar and sociodemographic variables like gender, education, marital status, and migrant status. Meanwhile, most studies like the National Comorbidity Survey Replication in the US (Wang et al., 2005) found that male gender was associated with lower odds of treatment along with other variables (married, low education, race/ethnic minority). In contrast to the US, neither Saudi Arabia nor Qatar showed any significant prediction of gender and other sociodemographic variables on lifetime treatment contact (Wang et al., 2005). Future studies could usefully explore reasons behind the country differences in interactions between socio‐demographic factors and treatment that could aid educational programs to focus on high‐risk groups.

### 5.7 Strengths and limitations

The study was based on a large and representative sample of population‐based adult Qataris and Arab residents living in Qatar during the time of the study using the CIDI version 3.3 as per DSM‐5 diagnostic criteria. The interviews were conducted over the phone therefore we were limited to those disorders that were likely to be of most public health importance, but could not cover them all. We decided for instance to omit conditions where reporting could be under‐declared such as substance use disorders and suicidality that carries severe legal consequences.

## 6 CONCLUSIONS

Our findings highlight a number of key issues of importance for mental health development and planning in Qatar and in countries that share similar demographic and social pressures. Lessons can also be learned about how to share best practice globally. For any service planning, identifying those at high risk is crucial. Our data highlights those at most risk of mental illness are younger cohorts, females, and Arab migrants of Qatar and therefore most likely to place the most burden on society. Cross‐country comparative data shows that one cannot assume that PTSD is confined to areas of conflict, but rather may represent response to more localized traumatic events in vulnerable people. Qatar appears to have some of the highest lifetime prevalence of mood or anxiety disorders and later median AOO than other WMH studies conducted in Arab countries. The overall lifetime treatment estimate reported here for Qatar were similar to neighboring Saudi Arabia but much lower than lifetime treatment rates reported in high‐income countries. Although proportion of any treatment contact in the year of onset was low in Qatar relative to high‐income countries they were consistent with or lower than those reported in some other Arab countries. Consistent with other studies, anxiety disorders had earlier AOO, greater delays for treatment contact, and lower projected lifetime treatment than mood disorders in Qatar. A high proportion of those with mental disorder who do seek help attend spiritual healers as well as general medical facilities (as opposed to specialized mental health services). The fact that most of those with bipolar disorder, a condition par excellence that requires intensive mental health intervention and monitoring, is not being seen by mental health services is an important gap that needs to be addressed. This need is particularly supported by the observations that bipolar disorder presents with the highest lifetime hospitalization, has a 5‐year delay in treatment onset, but is currently receiving the most lifetime treatment from spiritual healers as opposed to mental health services. The large proportion of those in treatment for mental disorder are being seen in non‐healthcare settings, another gap in service provision that needs addressing in the country.

## AUTHOR CONTRIBUTIONS

**Salma M. Khaled**: Conceptualization; formal analysis; data curation; methodology; investigation; supervision; project administration; writing—original draft; writing—review and editing; funding acquisition; resources. **Nour W. Z. Alhussaini**: Visualization; writing ‐ review and editing; software. **Majid Alabdulla**: Conceptualization; funding acquisition; project administration. **Nancy A. Sampson**: Conceptualization; formal analysis; writing—review and editing; methodology. **Ronald C. Kessler**: Conceptualization; writing—review and editing. **Peter W. Woodruff**: Conceptualization; funding acquisition; methodology; supervision; writing—review & editing. **Sheik Mohammed Al‐Thani**: Funding acquisition; resources; methodology; conceptualization; supervision; project administration; writing—review and editing.

## CONFLICT OF INTEREST STATEMENT

In the past 3 years, Dr. Kessler was a consultant for Cambridge Health Alliance, Canandaigua VA Medical Center, Holmusk, Partners Healthcare, Inc., RallyPoint Networks, Inc., and Sage Therapeutics. He has stock options in Cerebral Inc., Mirah, PYM, Roga Sciences and Verisense Health.

## ETHICS STATEMENT

Qatar University (QU‐IRB 1219‐EA/20) approved the study. The study's goal and methods were verbally explained to participants. Before each survey interview, consent to participate were verbally obtained using a phone script. All data were encrypted and saved on Qatar University's secure server. Each participant was assigned a case number and individual identifiers were retained in a password‐protected folder only available to the lead principle investigator, senior research assistant, and data analyst. All study researchers, including interviewers, signed confidentiality agreements preventing the sharing or use of participant personal information.

## Data Availability

The data that support the findings of this study are available from Dr. Salma M. Khaled, the principal investigator of the study at skhaled@qu.edu.qa, upon reasonable request and pending additional ethical approval.

## APPENDIX A

**TABLE A1:** Variation in the effects of cohort (age at interview) in predicting lifetime risk of DSM‐V/CIDI disorders in Qatar.

Disorder category	Age cohort	Sub‐samples					
		Early		Middle		Late	
		OR	95% CI	OR	95% CI	OR	95% CI
Panic disorder	18–29	14.8*	(5.3–41.8)	‐	‐	‐	‐
	30–39	5.8*	(2.1–15.7)	16.1*	(4.1–63.1)	‐	‐
	40–49	2.3	(0.8–6.7)	2.7	(0.8–8.7)	2.4	(0.5–11.0)
	50+	1.0	‐‐	1.0	‐‐	1.0	‐‐
	**Test statistics**	F_3,48_ (P_F_)	16.2 (<0.001)*	F_2,49_ (P_F_)	11.9 (<0.001)*	F_1,50_ (P_F_)	1.3 (0.251)
Generalized anxiety disorder	18–29	11.2*	(5.6–22.2)	‐	‐	‐	‐
	30–39	4.2*	(2.0–9.0)	7.4*	(3.1–17.3)	‐	‐
	40–49	1.8	(0.8–3.8)	3.3*	(1.7–6.5)	1.0	(0.3–4.1)
	50+	1.0	‐‐	1.0	‐‐	1.0	‐‐
	**Test statistics**	F_3,48_ (P_F_)	36.2 (<0.001)*	F_2,49_ (P_F_)	10.8 (<0.001)*	F_1,50_ (P_F_)	0.0 (0.948)
PCL‐SC PTSD^a^	18–29	5.1*	(2.8–9.3)	6.2*	(3.5–11.2)	‐	‐
	30–39	2.0*	(1.2–3.4)	4.8*	(3.1–7.6)	5.0*	(2.0–12.3)
	40–49	1.3	(0.7–2.3)	3.0*	(1.9–4.7)	2.0*	(1.0–3.8)
	50+	1.0	‐‐	1.0	‐‐	1.0	‐‐
	**Test statistics**	F_3,48_ (P_F_)	22.0 (<0.001)*	F_3,48_ (P_F_)	19.3 (<0.001)*	F_2,49_ (P_F_)	6.7 (0.003)*
Obsessive‐compulsive disorder^a^	18–29	6.1*	(2.1–17.7)	3.3	(0.7–16.2)	‐	‐
	30–39	4.6*	(1.7–12.1)	5.2*	(1.8–15.4)	4.0	(0.3–57.0)
	40–49	1.4	(0.5–4.3)	1.4	(0.3–6.0)	1.3	(0.4–4.6)
	50+	1.0	‐‐	1.0	‐‐	1.0	‐‐
	**Test statistics**	F_3,48_ (P_F_)	7.3 (<0.001)*	F_3,48_ (P_F_)	4.3 (0.009)*	F_2,49_ (P_F_)	0.5 (0.586)
Any anxiety disorders^a^	18–29	5.9*	(3.3–10.6)	4.1*	(2.3–7.5)	‐	‐
	30–39	2.6*	(1.5–4.6)	3.5*	(2.3–5.4)	5.2*	(2.4–11.3)
	40–49	1.4	(0.8–2.6)	2.2*	(1.4–3.3)	2.1*	(1.3–3.6)
	50+	1.0	‐‐	1.0	‐‐	1.0	‐‐
	**Test statistics**	F_3,48_ (P_F_)	29.0 (<0.001)*	F_3,48_ (P_F_)	13.3 (<0.001)*	F_2,49_ (P_F_)	10.7 (<0.001)*
Major depressive disorder	18–29	29.5*	(14.4–60.5)	6.1*	(2.3–16.1)	‐	‐
	30–39	9.2*	(4.4–19.2)	3.8*	(2.4–5.9)	16.8*	(6.3–45.2)
	40–49	2.6*	(1.1–6.0)	3.0*	(1.7–5.4)	2.5*	(1.3–4.7)
	50+	1.0	‐‐	1.0	‐‐	1.0	‐‐
	**Test statistics**	F_3,48_ (P_F_)	87.1 (<0.001)*	F_3,48_ (P_F_)	13.9 (<0.001)*	F_2,49_ (P_F_)	16.8 (<0.001)*
Broad bipolar disorder	18–29	7.5*	(3.8–14.6)	3.9*	(1.7–9.1)	‐	‐
	30–39	4.0*	(2.1–7.7)	4.5*	(2.4–8.4)	2.3	(1.0–5.3)
	40–49	3.4*	(1.6–7.1)	2.7*	(1.3–5.8)	1.3	(0.6–2.7)
	50+	1.0	‐‐	1.0	‐‐	1.0	‐‐
	**Test statistics**	F_3,48_ (P_F_)	16.1 (<0.001)*	F_3,48_ (P_F_)	7.3 (<0.001)*	F_2,49_ (P_F_)	2.6 (0.088)
Any mood disorders	18–29	18.2*	(11.4–29.0)	6.9*	(4.2–11.3)	‐	‐
	30–39	5.9*	(3.7–9.5)	5.3*	(3.4–8.4)	3.9*	(2.3–6.5)
	40–49	3.1*	(1.9–5.3)	3.0*	(1.9–4.7)	2.1*	(1.4–3.4)
	50+	1.0	‐‐	1.0	‐‐	1.0	‐‐
	**Test statistics**	F_3,48_ (P_F_)	140.3 (<0.001)*	F_3,48_ (P_F_)	25.0 (<0.001)*	F_2,49_ (P_F_)	15.7 (<0.001)*
Any disorders^a^	18–29	7.4*	(4.5–12.2)	4.5*	(2.6–7.6)	‐	‐
	30–39	3.0*	(1.8–5.1)	3.7*	(2.5–5.4)	3.2*	(1.7–6.2)
	40–49	1.8*	(1.1–3.1)	2.1*	(1.4–3.2)	1.9*	(1.2–3.2)
	50+	1.0	‐‐	1.0	‐‐	1.0	‐‐
	**Test statistics**	F_3,48_ (P_F_)	48.3 (<0.001)*	F_3,48_ (P_F_)	19.7 (<0.001)*	F_2,49_ (P_F_)	7.5 (0.001)*

*Note*: Results reflect weighted person‐year level data. Models include time intervals and gender as controls. Categorization of person‐years into early, middle, and late sub‐samples was based on cut‐points at the projected 33rd and 67th percentiles of onset risk for each mental disorder (Panic Disorder: 30/44; Generalized Anxiety Disorder: 28/43; PCL‐SC PTSD: 22/33; Obsessive‐Compulsive Disorder: 21/37; Any Anxiety Disorders: 21/33; Major Depressive Disorder: 25/36; Broad Bipolar Disorder: 20/28; Any Mood Disorders: 23/33; Any Disorders: 21/32). ‐ Excluded from the analysis because there were no person‐years from the age cohort with the disorder in the sub‐sample.

^a^ Disorder was only assessed in the Part 2 sample (*n* = 2583).

*Significant age difference at the 0.05 level. **p* < 0.05.

**TABLE A2:** Socio‐demographic predictors of lifetime treatment contact for the mood disorders.

Predictor	Major depressive disorder				Chi‐sq DF	*p*‐value	Broad bipolar disorder				Chi‐sq DF	*p*‐value
	Odds ratio	95% CI LB	95% CI UB	Chi‐sq			Odds ratio	95% CI LB	95% CI UB	Chi‐sq		
Age at interview												
18–29	4.9	1.4	17.7				1.4	0.4	4.5			
30–39	2.7	0.9	8.8				1.3	0.6	2.7			
40–49	2.2	0.9	5.6				1.2	0.5	2.7			
$\chi_{3}^{2}$				2.80*	3	0.050				0.19	3	0.906
Age at onset	1.5	1.0	2.3				2.0	1.2	3.2			
$\chi_{1}^{2}$				4.51*	1	0.039				7.36*	1	0.010
Female	1.1	0.8	1.5				1.0	0.6	1.6			
$\chi_{1}^{2}$				0.43	1	0.516				0.02	1	0.878
Education												
Student	0.9	0.5	1.6				0.6	0.2	1.5			
Low education	0.9	0.4	2.0				0.7	0.2	1.8			
Low‐average	1.1	0.7	1.7				1.3	0.6	2.7			
High‐average	0.6	0.3	1.2				0.7	0.2	2.0			
$\chi_{4}^{2}$				0.93	4	0.455				1.37	4	0.263
Ever married	1.1	0.6	1.7				1.1	0.6	1.9			
$\chi_{1}^{2}$				0.05	1	0.828				0.04	1	0.838
Migration	1.0	0.7	1.5				0.8	0.4	1.5			
$\chi_{1}^{2}$				0.00	1	0.974				0.63	1	0.432

*Note*: Based on a person‐year survival analysis controlling for AOO, time since onset, age at interview, sex, time‐varying education, time‐varying marital status, and time‐varying migration status, All treatment models used Part II weights. Analysis restricted to the subset responding to the long interview (*n* = 2583). Respondents who said they did talk to a medical doctor or other professional AND did not report their age of first treatment were excluded from the LT treatment analyses (*n* = 8).

**TABLE A3:** Socio‐demographic predictors of lifetime treatment contact for anxiety disorders.

Variables	Panic disorder					Generalized anxiety disorder					PCL‐SC PTSD					Obsessive‐compulsive disorder				
Predictor	OR	95% CI	*χ* ^2^	*χ* ^2^ *df*	*p*‐value	OR	95% CI	*χ* ^2^	*χ* ^2^ *df*	*p*‐value	OR	95% CI	*χ* ^2^	*χ* ^2^ *df*	*p*‐value	OR	95% CI	*χ* ^2^	*χ* ^2^ *df*	*p*‐value
Age																				
18–29	5.8	1.6–20.9				4.3	1.5–12.7				3.3	1.7–6.4				1.4	0.4–5.3			
30–39	4.5	1.2–16.7				2.4	0.9–6.1				1.8	1.0–3.2				0.6	0.2–1.4			
40–49	5.0	1.4–17.3				1.5	0.6–3.8				1.1	0.6–2.0				0.3	0.1–1.1			
$\chi_{3}^{2}$			2.7	3	0.072			3.15*	3	0.035			7.15*	3	0.001			4.79*	3	0.009
Age at onset	1.1	0.8–1.6				1.4	1.0–2.0				1.5	1.2–1.9				1.2	0.7–1.9			
$\chi_{1}^{2}$			0.56	1	0.462			3.43	1	0.071			13.21*	1	0.001			0.51	1	0.479
Female	0.9	0.5–1.7				0.9	0.6–1.4				1.1	0.8–1.5				1.4	0.8–2.5			
$\chi_{1}^{2}$			0.14	1	0.708			0.07	1	0.797			0.26	1	0.612			1.36	1	0.254
Education																				
Student	1.4	0.4–4.1				0.7	0.3–1.7				0.8	0.5–1.4				0.3	0.2–0.6			
Low education	0.6	0.2–1.8				1.1	0.5–2.6				0.8	0.5–1.3				0.4	0.2–0.8			
Low‐average	1.1	0.5–2.5				0.9	0.5–1.7				1.1	0.8–1.6				0.4	0.2–0.8			
High‐average	0.6	0.2–2.3				0.3	0.1–0.9				0.9	0.4–2.0				0.5	0.1–2.3			
$\chi_{4}^{2}$			0.71	4	0.597			1.2	4	0.326			0.70	4	0.597			6.64*	4	0.001
Ever married	1.7	0.8–3.6				1.2	0.6–2.5				1.2	0.8–1.8				1.0	0.5–2.1			
$\chi_{1}^{2}$			1.87	1	0.184			0.25	1	0.617			0.55	1	0.464			0.00	1	0.955
Migration	1.4	0.7–2.6				1.1	0.7–1.7				1.0	0.7–1.4				0.7	0.4–1.1			
$\chi_{1}^{2}$			1.07	1	0.311			0.07	1	0.793			0.04	1	0.848			2.60	1	0.118

*Note*: Based on a person‐year survival analysis controlling for AOO, time since onset, age at interview, sex, time‐varying education, time‐varying marital status, and time‐varying migration status, All treatment models used Part II weights. Analysis restricted to the subset responding to the long interview (*n* = 2583).Respondents who said they did talk to a medical doctor or other professional AND did not report their age of first treatment were excluded from the LT treatment analyses (*n* = 8).

**p* < 0.05.
